# Robust immunoscore model to predict the response to anti-PD1 therapy in melanoma

**DOI:** 10.18632/aging.102556

**Published:** 2019-12-03

**Authors:** Run-Cong Nie, Shu-Qiang Yuan, Yun Wang, Ying-Bo Chen, Yan-Yu Cai, Shi Chen, Shu-Man Li, Jie Zhou, Guo-Ming Chen, Tian-Qi Luo, Zhi-Wei Zhou, Yuan-Fang Li

**Affiliations:** 1 Department of Gastric Surgery and Melanoma Surgical Section, Sun Yat-sen University Cancer Center, State Key Laboratory of Oncology in South China, Collaborative Innovation Center for Cancer Medicine, Guangzhou, China; 2 Department of Hematologic Oncology, Sun Yat-sen University Cancer Center, State Key Laboratory of Oncology in South China, Collaborative Innovation Center for Cancer Medicine, Guangzhou, China; 3 VIP Department, Sun Yat-sen University Cancer Center, State Key Laboratory of Oncology in South China, Collaborative Innovation Center for Cancer Medicine, Guangzhou, China; 4 Department of Gastric Surgery, The 6th Affiliated Hospital, Sun Yat-sen University, Guangzhou, China; 5 Department of Experimental Research (Cancer Institute), Sun Yat-sen University Cancer Center, State Key Laboratory of Oncology in South China, Collaborative Innovation Center for Cancer Medicine, Guangzhou, China

**Keywords:** melanoma, PD1, immunoscore, response, CIBERSORT

## Abstract

This study aimed to construct immune-related predictors to identify responders to anti-PD1 therapy of melanoma through CIBERSORT algorithm. Using the least absolute shrinkage and selection operator (LASSO) logistic regression, we constructed an immunoscore consisting of 8 immune subsets to predict the anti-PD1 response. This score achieved an overall accuracy of AUC = 0.77, 0.80 and 0.73 in the training cohort, validation cohort and on-anti-PD1 cohort, respectively. Patients with high immunoscores had significantly higher objective response rates (ORRs) than did those with low immunoscores (ORR: 53.8% vs 17.7%, P < 0.001 for entire pre-anti-PD1 cohort; 42.1% vs 15.1%, P = 0.022 for on-anti-PD1 cohort; 66.7% vs 16.7%, P = 0.038 for neoadjuvant anti-PD1 cohort). Prolonged survival trends were observed in high-immunoscore group (1-year PFS: 42.4% vs 14.3%, P = 0.059; 3-year OS: 41.5% vs 31.6%, P = 0.057). Furthermore, we found that high-immunoscore group exhibited higher fractions of tumor-infiltrating lymphocytes and an increased IFN-γ response. Analysis of the results of the GSEA indicated a significant enrichment of antitumor immunity pathways in the high-immunoscore group. Therefore, this study indicated that we constructed a robust immunoscore model to predict the anti-PD1 response of metastatic melanoma and the neoadjuvant anti-PD1 response of resectable melanoma.

## INTRODUCTION

The improved understanding of the immune checkpoint and the application of its inhibitors in cancer immunotherapy has dramatically improved the survival outcomes of metastatic melanoma [1, 2]. The novel drugs that block the binding of programmed death 1 receptor (PD1) to its ligand, PD1 ligand 1 (PDL1), have increased the historically median overall survival (OS) of advanced melanoma from approximately 8 months to over 57 months [3–5]. However, despite this tremendous advancement, only a subset of patients with metastatic melanoma receiving PD1 inhibitors derives clinical benefit [6]; moreover, anti-PD1 therapies, especially combination therapeutic strategies, are correlated with severe immune-related adverse events (irAEs) and could be very costly. Thus, there exists an interesting issue to identify effective biomarkers to predict the response to anti-PD1 therapy.

PD1 inhibitors exert antitumor efficacy by reinvigorating dysfunctional or exhausted T cells [7]. Several studies have reported that a special subset of T cells, such CD8^+^ TCF7^+^ T cells [8], strongly correlated with the response to anti-PD1 therapy in melanoma. Furthermore, the signatures of the T cell repertoire that included IFN-γ responses [9] as well as those signatures representing the activation, exhaustion and cytotoxicity of T cells [10, 11] were reported to have associations with the anti-PD1 response. Mechanistically, other immune subsets within the tumor microenvironment (TME) beyond T cells, such as macrophages, natural killer (NK) cells and even eosinophils, may also affect anti-PD1 efficacy [6, 12]. Nonetheless, how and which of these immune subsets modulate the PD1 inhibitor-mediated activity in melanoma remains poorly understood and should be urgently clarified.

To comprehensively profile the immune landscape of the TME of melanoma patients treated with PD1 inhibitors, we used the CIBERSORT algorithm [13, 14] to enumerate the fractions of 22 immune cell subsets based on RNA gene expression profiles and used the least absolute shrinkage and selection operator (LASSO) logistic regression to establish an immunoscore model to predict anti-PD1 efficacy.

## RESULTS

### Patient characteristics

After rigid filter criteria (Supplementary Figure 1), a total of six series were finally analyzed; these series included five GEO datasets [10, 11, 15–17] (GSE115821, GSE123728, GSE78220, GSE91061 and GSE93157) and one TCGA dataset, comprising 691 melanoma patients. Table 1 shows detailed patient characteristics of the included series. The median age at diagnosis was 50.0 (range: 38.0-85.0) years, and 342 (49.5%) of the patients were male. A total of 228 (33.0%) patients were treated with anti-PD1 therapy, among which 136 biopsies were obtained before anti-PD1 therapy (pre-anti-PD1 therapy cohort), and the remaining patients were obtained during anti-PD1 therapy (on-anti-PD1 therapy cohort); the overall ORR was 26.8% (61/228).

**Table 1:** Clinical characteristics of the patients.

	**No. of patients (n = 691) (%)**
Series	
GSE115821	37 (5.3)
GSE123728	24 (3.5)
GSE78220	28 (4.1)
GSE91061	109 (15.8)
GSE93157	25 (3.6)
TCGA	468 (67.8)
Age	
median, range	50.0 (38.0-85.0)
Gender	
Male	342 (49.5)
Female	203 (29.4)
Unknown	146 (21.1)
TNM stage	
I/II	219 (31.7)
III	194 (28.1)
IV	222 (32.1)
Unknown	56 (8.1)
Anti-PD-1 therapy sample	
No	463 (67.0)
Yes	228 (33.0)
Response to anti-PD-1 therapy	
Response	61 (26.8)
No response	165 (72.3)
Unknown	2 (0.9)

TCGA, the Cancer Genome Atlas; PD-1, Programmed cell death protein 1.

### Construction of the immunoscore model

**Figure 1:**
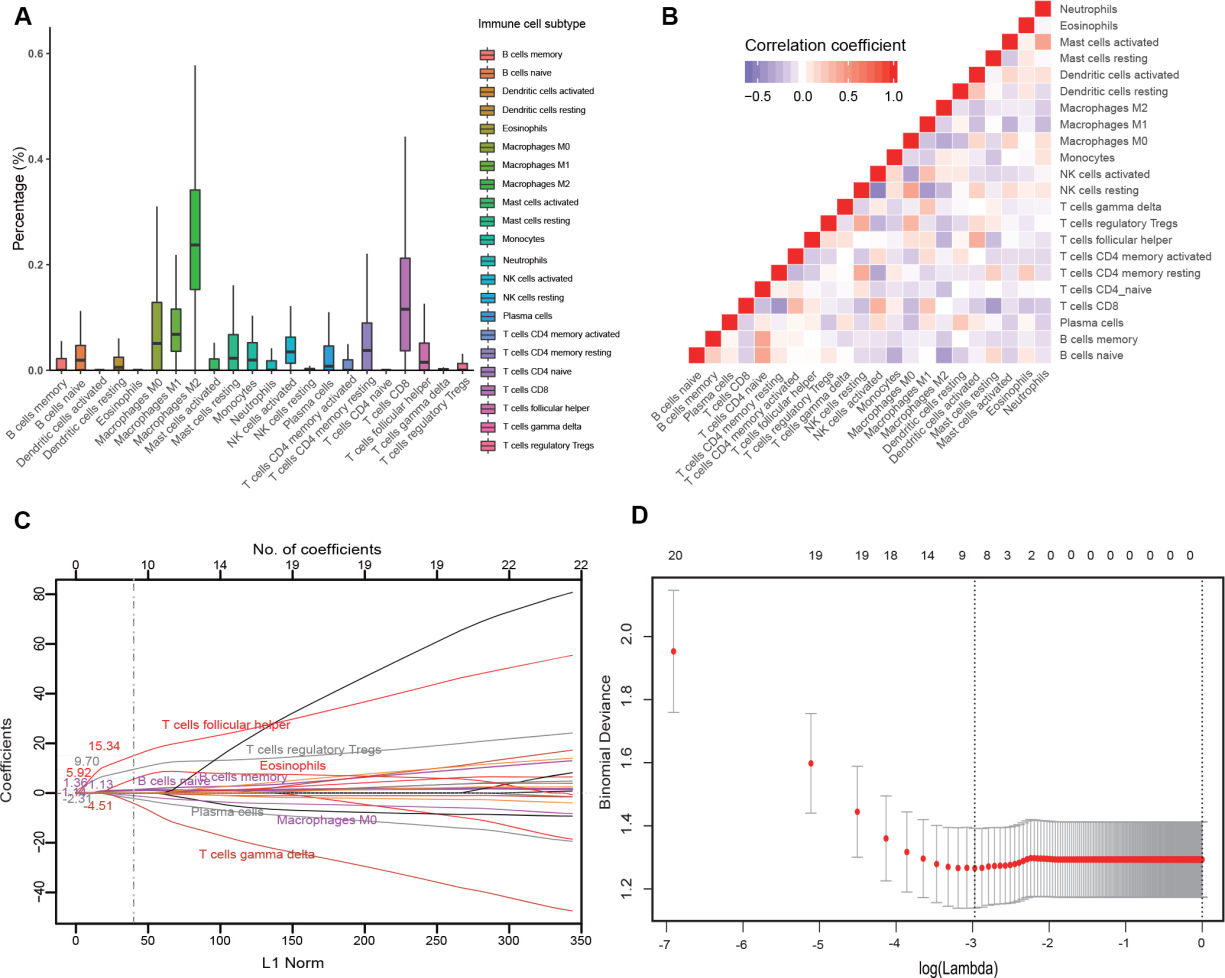
**Construction of the immunoscore model.** (**A**) Bar charts summarizing the fractions of 22 immune cell subsets of 134 melanoma tissues before anti-PD1 therapy. (**B**) Hierarchical clustering shows the collinearity of 22 immune cell subsets in the training cohort, where each cell indicates the Pearson correlation between the row and column corresponding immune cell subsets. The legend characterizes the color change corresponding to the change of correlation coefficient from -0.65 to 1.0. (**C**) LASSO coefficient of the 22 immune cell subsets. Each curve corresponds to an immune cell subset; the dotted line indicates the value of λ chosen by 200-fold cross-validation via min criteria. (**D**) 200-fold cross-validation for variable selection in the LASSO regression. PD1, programmed death 1; LASSO, least absolute shrinkage and selection operator.

Among the 22 immune cell subsets, M2 macrophages, CD8^+^ T cells, M1 macrophages, M0 macrophages and CD4^+^ memory resting T cells were the five most abundant immune cell fractions, the sum of which was more than 65% (Figure 1A). In the training cohort, we observed weak to strong correlations (r: -0.52 - 0.43) among the fractions of the 22 immune cell subsets (Figure 1B), which would bias the results of traditional logistic regression. Therefore, we applied LASSO logistic regression to select parameters to predict the response to anti-PD1 therapy (Figure 1C, 1D), and eight immune subsets were finally used to construct an immunoscore in the training cohort. The formula for the immunoscore of each patient is that: immunoscore = (1.13 × fraction level of naive B cells) + (1.36 × fraction level of memory B cells) + (5.92 × fraction level of eosinophils) + (9.70 × fraction level of follicular helper T cells) + (15.34 × fraction level of Tregs) - (1.14 × fraction level of M0 macrophages) - (2.31 × fraction level of plasma cells) - (4.52 × fraction level of γδT cell). Based on the fractions of eight immune subsets, we calculated the immunoscore of the total population. The median immunoscore of the total population (691 patients) was 0.38 (range: -0.93 - 3.16).

### Evaluation of the immunoscore model

**Figure 2:**
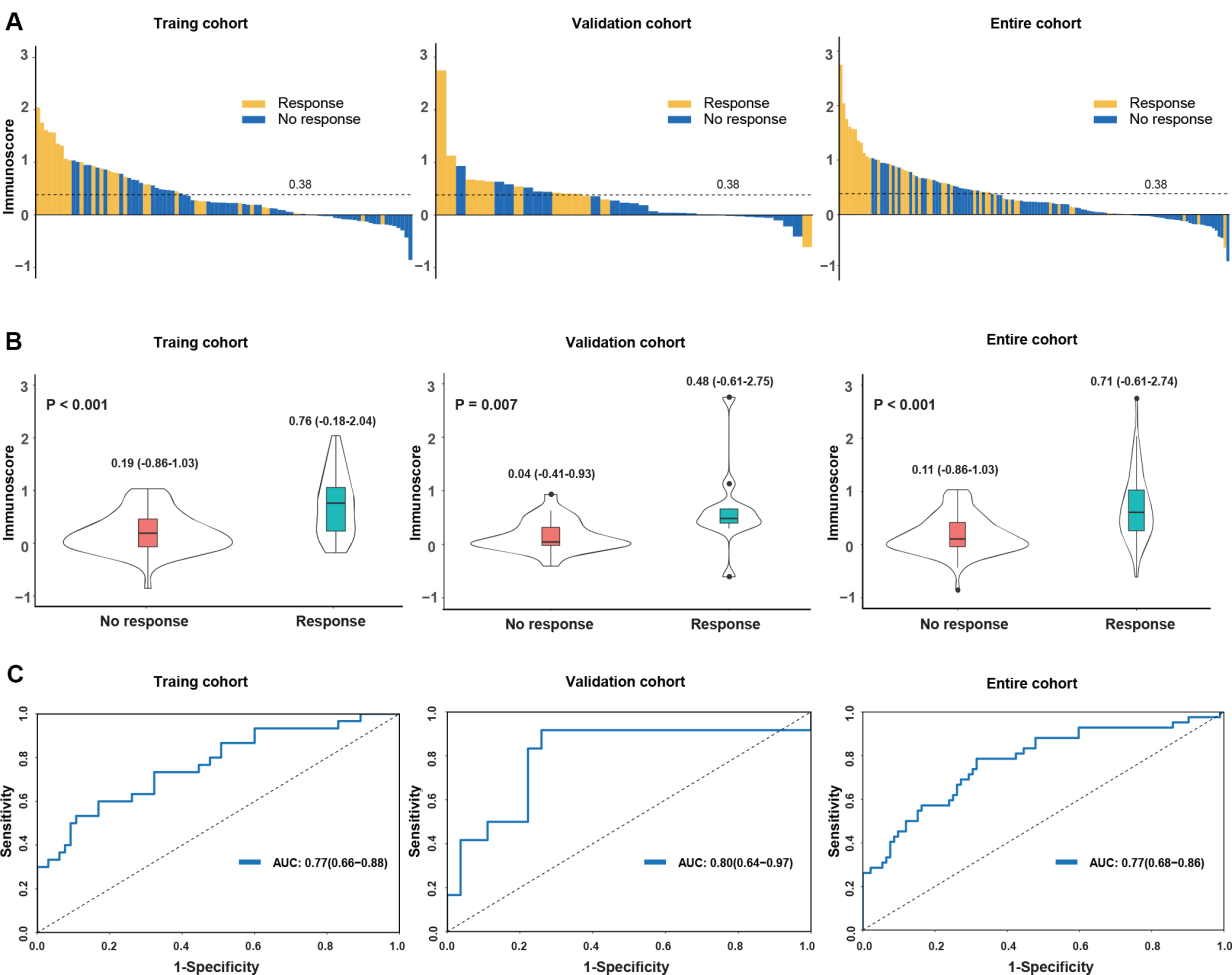
**Distribution of the immunoscore and response status to anti-PD1 therapy in the training, validation and entire cohorts.** (**A**) Waterfall plots for the distribution of the immunoscore and response status of individual patients. (**B**) Distribution of the immunoscore in responders and nonresponders. The box plots inside the violin indicate the median value and interquartile range of the immunoscore. We calculated the P-value with a one-way ANOVA test. (**C**) Receiver operating characteristic (ROC) curves of the immunoscore in three cohorts. The area under the ROC curve in the training, validation and entire cohorts was 0.77, 0.80 and 0.77, respectively.

We then investigated the predictive value of the immunoscore to anti-PD1 response in the training cohort, validation cohort, entire cohort and on-anti-PD1 therapy cohort. The correlation between the distribution of immunoscores and the response status showed that patients with high immunoscores generally had better responses to anti-PD1 therapy than did those with low immunoscores (Figure 2A, Supplementary Figure 2A). The immunoscore of the responders was significantly higher than those of the nonresponders (0.76 vs 0.19, P < 0.001 for the training cohort; 0.48 vs 0.04, P = 0.007 for the validation cohort; 0.71 vs 0.11, P < 0.001 for the entire cohort; 0.23 vs 0.09, P < 0.001 for the on-anti-PD1 cohort) (Figure 2B,Supplementary Figure 2B). The prognostic value of the immunoscore was evaluated using ROC analysis, with AUCs of 0.77 (0.66-0.88), 0.80 (0.64-0.97), 0.77 (0.68-0.86) and 0.73 (0.59-0.87) in the training, validation, entire cohort (Figure 2C) and on-anti-PD1 cohort (Supplementary Figure 2C), respectively. These results indicated that the immunoscore could effectively predict the response to anti-PD1 therapy.

**Figure 3:**
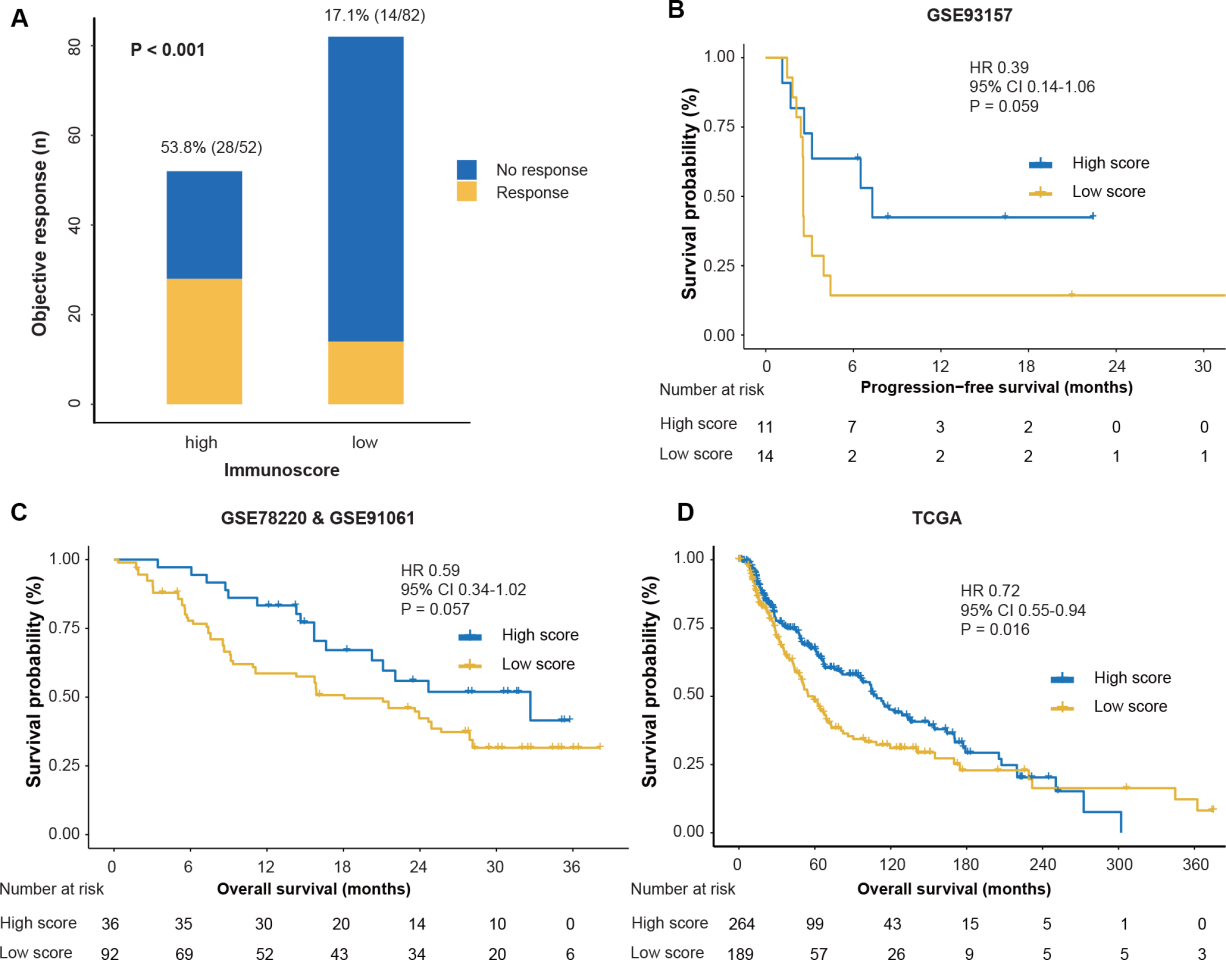
**Response and survival outcomes between high- and low-immunoscore groups.** (**A**) Objective response rate between high- and low-immunoscore groups across the pre-anti-PD1 melanoma datasets. “Pre” indicates the biopsy before anti-PD1 therapy. We calculated the P-value with the χ^2^ test. (**B**) Comparison of PFS between high- and low-immunoscore groups in the GSE93157 dataset. (**C**) Comparison of the OS between high- and low-immunoscore groups in the GSE78220 and GSE91061 datasets. (**D**) Comparison of the OS between high- and low-immunoscore groups in the TCGA dataset. Hazard ratios (HR) and P-values were calculated using the Cox regression analysis and log-rank test; all statistical tests were two-sided. PD1, programmed death-1; OS, overall survival; PFS, progression-free survival; TCGA, The Cancer Genome Atlas.

With the immunoscore formula, the total population was divided into high- and low-immunoscore groups according to the median value (0.38). The objective response rate (ORR) was 53.8% (28/52) in the high-immunoscore group and 17.7% (14/82) in the low-immunoscore group (P < 0.001) for the entire pre-anti-PD1 cohort (Figure 3A). Consistent results were observed for the on-anti-PD1 cohort (ORR: 42.1% [8/19] vs 15.1% [11/73], P = 0.022; Supplementary Figure 2D). We then investigated the prognostic value of the immunoscore to predict survival outcomes. The GSE93157 series reported the PFS of patients treated with anti-PD1 therapy. The 1-year PFS was 42.4% (95% confidence interval (CI) 20.6%-87.2%) in the high-immunoscore group and 14.3% (95% CI 4.0%-51.5%) in the low-immunoscore group; the PFS difference showed a robust trend toward significance (HR 0.39, 95% CI 0.14-1.06; P = 0.059; Figure 3B). The GSE78220 and GSE91061 series reported the OS outcomes of patients treated with anti-PD1 therapy. We noted that patients in the high-immunoscore group had a longer OS trend than did those in the low-immunoscore group (3-year OS: 41.5% [23.7%-72.7%] vs 31.6% [22.9%-43.5%]; HR 0.59, 95% CI 0.34-1.02; P = 0.057; Figure 3C).

Next, we explored the performance of immunoscore in the non-anti-PD1 therapy cohort using the TCGA dataset and found that patients in the high-immunoscore group were associated with prolonged OS (HR 0.72, 95% CI 0.55-0.94; P = 0.016; Figure 3D). The multivariate Cox regression analysis demonstrated that, adjusting for covariates of gender, age and TNM stage, the immunoscore remained the prognostic factor for OS (HR 0.63, 95% CI 0.47-0.85; P = 0.002; Supplementary Table 1).

### Correlation between immunoscore with clinical and molecular features

**Figure 4:**
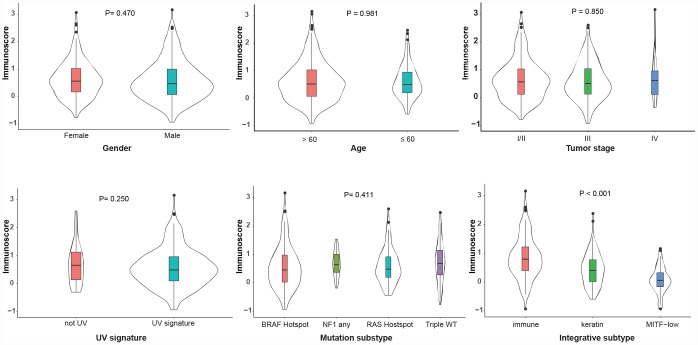
**Distribution of the immunoscore in different clinicopathological characteristics in the TCGA dataset.** The classifications of the UV signature, mutation subtype and integrative subtype are described by the TCGA genomic classification program [18]. The box plots inside the violin indicate the median value and interquartile range of immunoscore. We calculated the P-values using one-way ANOVA. UV, ultraviolet; TCGA, The Cancer Genome Atlas.

**Figure 5:**
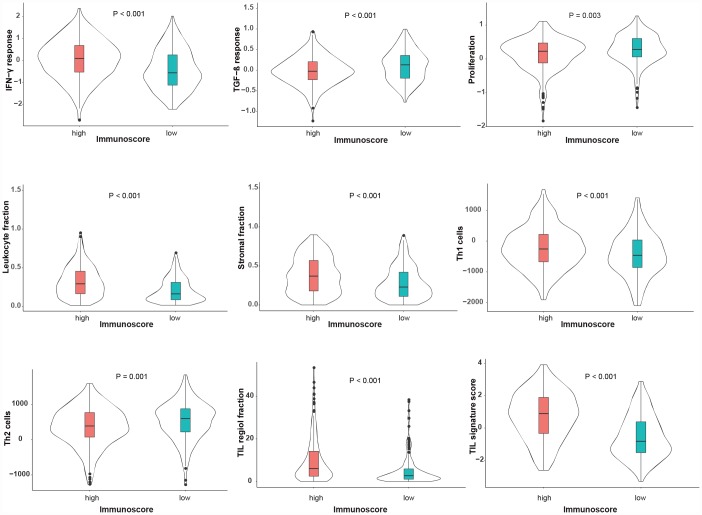
**Immune-related features between high- and low-immunoscore groups in the TCGA dataset.** These immune-related features are described according to the immune classification study of Thorsson et al. [34]. The P-values were calculated using one-way ANOVA.

The correlations between immunoscore with clinical and molecular features were investigated in the TCGA dataset. As shown in Figure 4, the immunoscore was not different, regardless of gender, age, TNM stage, UV signature status or the mutation subtype (all P > 0.1). Interestingly, regarding the integrative subtype, which was classified according to the TCGA genomic classification program [18], the immune subtype was associated with the highest immunoscore, following by the keratin and MITF-low subtype (P < 0.001). Subsequently, we explored the association between immunoscore and immune-related responses and found that the high-immunoscore group exhibited an increased antitumor immune response and higher fractions of tumor-infiltrating lymphocytes (TILs) (Figure 5), suggesting that the immunoscore can indeed reflect the immune level of melanoma.

### Biological pathways associated with the immunoscore model

**Figure 6:**
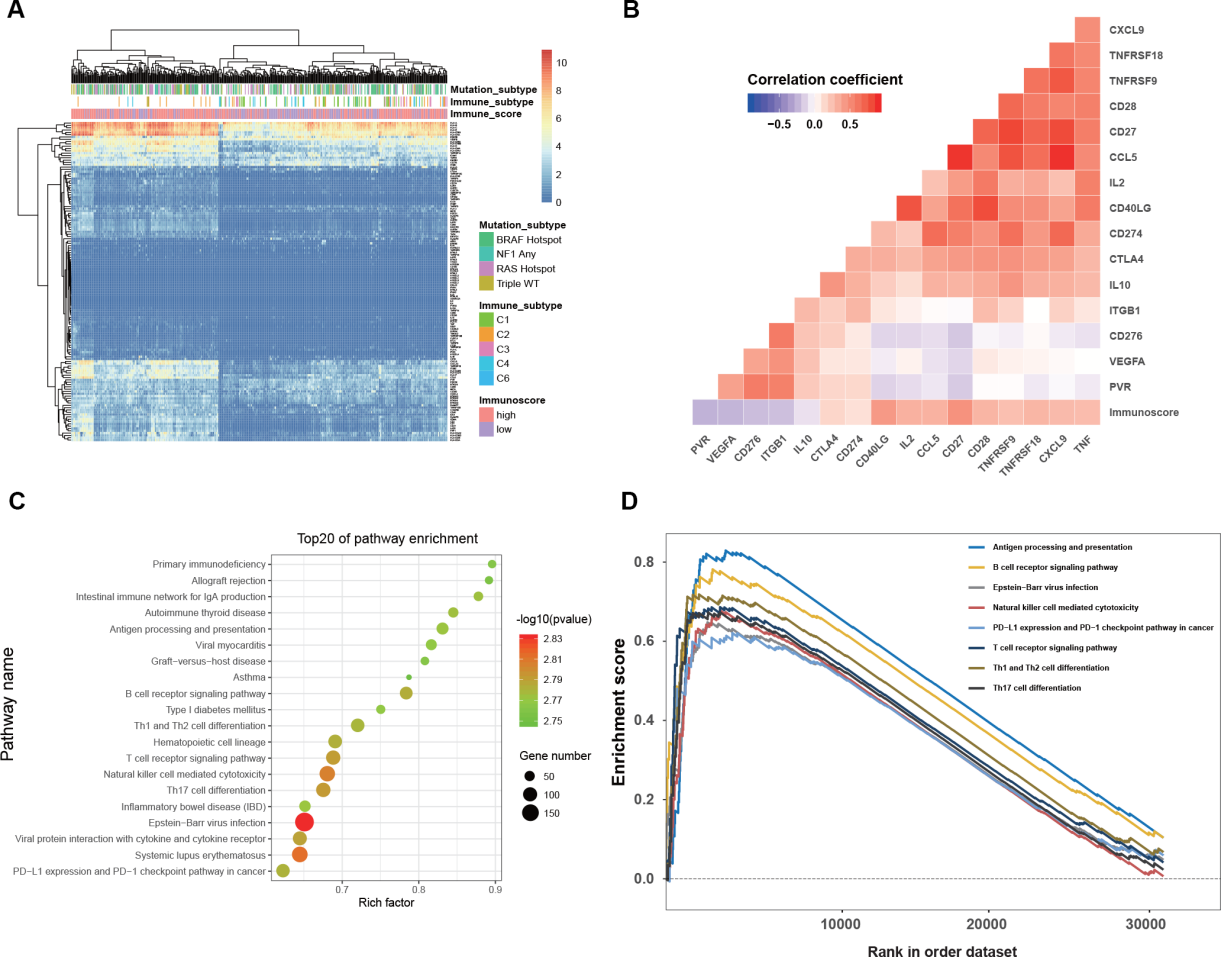
**Clinical significance and biological function of the immunoscore.** (**A**) Hierarchical clustering of 138 immune-related gene in 468 melanoma patients from the TCGA dataset. (**B**) Correlation matrix of immunoscore and the expression of certain immune-related genes. The color of each cell indicates the value of the corresponding Pearson correlation coefficients. (**C**) Bubble plot of the top 20 biological pathways and processes enriched in the high-immunoscore group using the gene set of “c2.cp.kegg.v6.1.symbols”. The legend shows the values of gene number and -log 10- transformed P-values; all P-values < 0.001. (**D**)Gene set enrichment analysis reveals the 8 antitumor immune pathways enriched in the high-immunoscore group.

The gene expression of the TCGA dataset was analyzed to explore the potential biological process related to the immunoscore. The hierarchical clustering of 138 immune- or antigen presentation-related gene levels (detailed genes list shown in Supplementary Table 2) indicated that patients clustered better with immunoscore than with immune subtype and mutation subtype (Figure 6A), and the immunoscore was significantly associated with the immune checkpoint genes (all P < 0.001, Figure 6B). Finally, the GSEA was performed to elaborate the biological phenotypes of the immunoscore model. The top 20 GSEA pathways enriched in high-immunoscore were mainly immune related (all P < 0.002, Figure 6C), eight of which were the antitumor immunity pathways, including antigen process, B/T cell receptor signaling, Epstein-Barr virus infection, NK cell-mediated cytotoxicity, PD-L1 pathway, Th1, Th2 and Th7 cell differentiation (Figure 6D).

## DISCUSSION

The therapeutic landscape of advanced melanoma has dramatically shifted from cytotoxic drugs to BRAF-/ MEK-targeted agents and, recently, PD1 inhibitors [2], resulting in a durable response and prolonged survival. Nonetheless, innate resistance and progression after the initial clinical response (acquired resistance) remain the major issue [19] to be resolved, and it is urgent to predict the beneficiaries before or during anti-PD1 therapy. By profiling the 22 immune cell phenotypes before and during therapy in melanoma patients treated with PD1 blockades, we identified several immune subsets that were associated with the anti-PD1 response. An immunoscore model was established to predict the response effectively; the robustness of the model was verified by a series of molecular features and biological pathway exploration.

Among the features of TME, the immune cell phenotype is one of the critical keys to predict the response to anti-PD1 therapy. The baseline level and location of CD8^+^ T cells in pretreatment tumor biopsies have been reported to correlate with an effective anti-PD1 response [20–22]. Furthermore, through high-dimensional single-cell RNA-seq, Sade-Feldman et al. [8] found that a subset of CD8^+^ T cells that express the transcription factor TCF7 protein can better predict the clinical anti-PD1 response. Beyond T cells, the numerous other types of immune cells (e.g., myeloid cells and NK cells) in the TME intertwine and interact in a highly orderly manner that can also affect anti-PD1 efficacy [6, 23]. In line with this notion, we found that the adverse macrophage types (M0, M1 and M2) and the favorable T cell subsets (CD8^+^ T cell and CD4^+^ memory resting T cell) account for a major fraction of the immune cells, and we noted the strong association among the fractions of the 22 immune cell subsets; these results further indicated that the features of the TME in melanoma are complicated and coordinated.

The central finding of our study is that we constructed a robust immunoscore model that could effectively predict clinical responses and survival outcomes to anti-PD1 therapy. Anti-PD1 therapy revitalizes the pre-existing tumor immune response [6, 11, 24]. Therefore, biomarkers that represent pre-existing tumor immune phenotypes can theoretically be used to predict the anti-PD1 response. We noted that patients with high immunoscores have a significantly higher proportion of TILs, which increases the IFN-γ response and reduces the TFG-β response. Recent studies have demonstrated that the increased IFN-γ signature is a robust indicator of reinvigorated T cells and may improve the response likelihood to anti-PD1 therapy [9, 25]. The GSEA results of our study showed that eight of the top 20 biological pathways enriched in the high-immunoscore population were antitumor immunity related. Therefore, these results indicated that the immunoscore can reflect the pre-existing tumor immune response, and we can distinguish the responders from nonresponders before the initial use of PD1 blockade through the immunoscore, which can avoid the unnecessary use of anti-PD agents for metastatic melanoma.

Therapeutic combinations, including PD1 inhibitors in combination with innate immune stimulants [26] or molecularly targeted agents, are promising strategies to enhance the efficacy and reduce the risk of irAEs. Nonetheless, routine tests of PDL1 expression or lactate dehydrogenase are not predictive biomarkers [3–5], and these combinational strategies still lack appropriate biomarkers [27]. Surprisingly, our study showed that the immunoscore positively correlated with several co-stimulating molecules, such as 4-1BB (TNFRSF9) and GITR (TNFRSF18), suggesting that the immunoscore may serve as a potential biomarker to assist in the identification of the beneficiaries for these agonistic antibodies in combination with anti-PD1 inhibitors, which deserves further exploration.

The dynamic risk assessment using serial tumor biomarkers may promote the accurate prediction of clinical outcomes [28]. Riaz et al. reported that the change in CD8^+^ T cells, NK cells and M1 macrophages during Nivo therapy correlated with the response to therapy [11]. Similarly, in addition to immunoscore pre-therapy, we found that immunoscore on-therapy can also predict anti-PD1 efficacy. Therefore, we inferred that a comprehensive analysis of serial immunoscores in different treatment stages can improve the prediction accuracy of the anti-PD1 response; this concept should be further explored.

Recently, two clinical studies have reported that neoadjuvant anti-PD1 therapy is associated with encouraging clinical, pathological complete response (pCR) and survival outcomes [16, 29]. Nonetheless, PD1 inhibitors might exert an antitumor effect in a delayed manner and in some cases, could result in “tumor flare” or in the appearance of new metastases [30]; thus, there is concern [31, 32] for whether neoadjuvant PD1 blockade is appropriate for melanoma and how to predict the response before its initial use? Notably, we found that the immunoscore is independent of tumor stage. Moreover, for the neoadjuvant anti-PD1 cohort (GSE123728) [16], the ORR was 66.7% (8/12) in the high-immunoscore group and 16.7% (2/12) in the low-immunoscore group (P = 0.038), indicating that immunoscore is also a valid biomarker for neoadjuvant PD1 blockade. Therefore, it is scientifically rational that we could use immunoscore to identify the responders to neoadjuvant PD1 blockade and guide the longer treatment duration to increase the likelihood of achieving a pCR, which may decrease the extent of surgery and prolong survival [33].

This study has several limitations. First, this study was based on publicly available datasets; thus, we could not obtain all the clinicopathological characteristics for each patient in the GEO datasets. However, the characteristics, mutation subtype and immune-related response of the patients in the TCGA dataset provided preliminary evidence to explore the mechanism of immunoscore. Another limitation in the study interpretation was that we cannot evaluate the immunoscore in the core of the tumor and the invasive margin separately since the gene expression profiles were derived from a core sample of tumor tissue. Third, the number of the patients receiving anti-PD1 therapy in our study is not large (226 subjects). Therefore, the immunoscore algorithm should be optimized with larger population. Finally, all datasets were obtained from retrospective studies, and potential bias due to the unbalanced clinicopathological features cannot be neglected. Further prospective studies are still required to validate this immunoscore model.

## CONCLUSIONS

Taken together, these results indicated that we constructed a robust immunoscore model to predict the anti-PD1 response of metastatic melanoma and the neoadjuvant anti-PD1 response of resectable melanoma.

## MATERIALS AND METHODS

### Search strategy and series collection criteria

In July 2019, we conducted a systematic search of Gene Expression Omnibus (GEO) datasets (https://www.ncbi.nlm.nih.gov/geo/) to identify the melanoma expression data treated with anti-PD1 therapy. GEO search terms are shown in Appendix S1 (supplementary materials). We also downloaded the RNA-sequencing (RNA-seq) data of the SKCM cohort from The Cancer Genome Atlas (TCGA) (https://xenabrowser.net/datapages/) to further explore the molecular features and survival outcomes. The inclusion and exclusion criteria for series collection and analysis protocols are shown in Supplementary Figure 1. Two independent authors (RCN and SQY) were responsible for assessing the potential eligible series, and the discrepancies during the series search and data extraction were resolved by two senior authors (ZWZ and YFL).

### Data collection and processing

We downloaded the series matrix files from the corresponding GEO dataset website and retrieved the relevant clinical data and expression data for each GEO dataset using the GEOquery package of R software. For any series matrix that was not available through the GEOquery package, we downloaded the matrix files from the supplementary file of the relevant GEO dataset website. For the GES91061 dataset, we downloaded the survival information from supplementary material in the corresponding study [11]. The relevant clinical data and RNA-seq of TCGA were downloaded from the xenabrowser website (https://xenabrowser.net/datapages/), and the molecular features were extracted from the supplementary materials of the study of Thorsson et al. [34]. All integrated clinicopathological characteristics of the GEO and TCGA are provided in Appendix S2 (supplementary materials). We applied the quantile method and trans per million (TPM) method to normalize the expression between microarray data (limma package) [35] and RNA-seq data, respectively.

### Estimation of immune cell subsets

The CIBERSORT method with an LM22 gene signature matrix that contains 547 genes was used to estimate the immune cell subsets. The standardized processed expression data with relevant annotation were uploaded to the CIBERSORT website (http://cibersort.stanford.edu/), and the LM22 gene signature with 200 permutations was used to run the CIBERSORT algorithm; the final CIBERSORT output contains the fractions of 22 hematopoietic immune subsets, including seven T cell types, B cell types (activated and resting), plasma cells, natural killer (NK) cells and myeloid cells [13, 14].

### Random grouping method

To estimate the immunoscore model to predict the response of anti-PD1 therapy, we used the stratified randomization approach to split the pre-anti-PD1 melanoma dataset into training and validation cohorts at a ratio of 7:3 through the “rsample” package. The patients in the training cohort were used to identify the predictors to construct the model, which was then validated by the remaining 30% of patients and the on-anti-PD1 therapy melanoma patients as the validation cohorts.

### Clinical outcomes

The primary outcome is the objective response rate (ORR) to anti-PD1 therapy, which was defined as the proportion of the complete or partial responses. OS was defined as the date of diagnosis to death from any cause. Progression-free survival (PFS) was defined as the date of diagnosis to progressive disease or death from any cause.

### Statistical analysis

One-way ANOVA and χ^2^ test were used to compare the continuous and categorical variables, respectively. We applied Pearson’s correlation test with correlation coefficient ® to quantify the correlations among the immune cell subsets and between the immunoscore and specific gene expression. The penalized LASSO logistic regression (“glmnet” package) [36] was used to select reliable predictors of anti-PD1 therapy among the 22 immune cell subsets. The optimal values of the penalty parameter λ were determined by 200-time cross-validation via the min criteria. Then, we constructed an immunoscore model based on the fraction of selected immune subsets using the logistic regression coefficients in the training cohort. The predictive values of the immunoscore to anti-PD1 response were depicted by a receiver operating characteristics (ROC) curve and quantified by the area under the curve (AUC) of ROC using the “ROCit” package [37]. Survival was estimated by the Kaplan-Meier method and compared using the log-rank test. A Cox regression model was used to determine prognostic performance. Gene set enrichment analysis (GSEA) was performed to identify the biological pathways and processes using the “c2.cp.kegg.v6.1.symbols” gene set [38, 39].

All statistical tests were two-sided with P-values < 0.05 considered significant, and all statistical analyses were performed using R version 3.6.0 (http://www.r-project.org).

## Supplementary Material

Supplementary Appendix

Supplementary Figures

Supplementary Table 1

Supplementary Table 2
