# Supplementary material for: Robust immunoscore model to predict the response to anti-PD1 therapy in melanoma
Source: Aging (Albany NY). 2019 Dec 3;11(23):11576–90. doi: 10.18632/aging.102556 (PMC6932919; doi:10.18632/aging.102556)
Supplement: Supplementary Appendix [file aging-11-102556-s002..pdf]

Supplementary Appendix. GEO search terms (n = 113)

(Melanoma [Title])) AND "Homo sapiens"[porgn:_txid9606] AND (("Expression profiling by high throughput sequencing"[Filter] OR "Expression profiling by array"[Filter]) AND "attribute name tissue"[Filter]).
