# Supplementary material for: Robust immunoscore model to predict the response to anti-PD1 therapy in melanoma
Source: Aging (Albany NY). 2019 Dec 3;11(23):11576–90. doi: 10.18632/aging.102556 (PMC6932919; doi:10.18632/aging.102556)
Supplement: Supplementary Figures [file aging-11-102556-s004..pdf]

SUPPLEMENTARY FIGURES

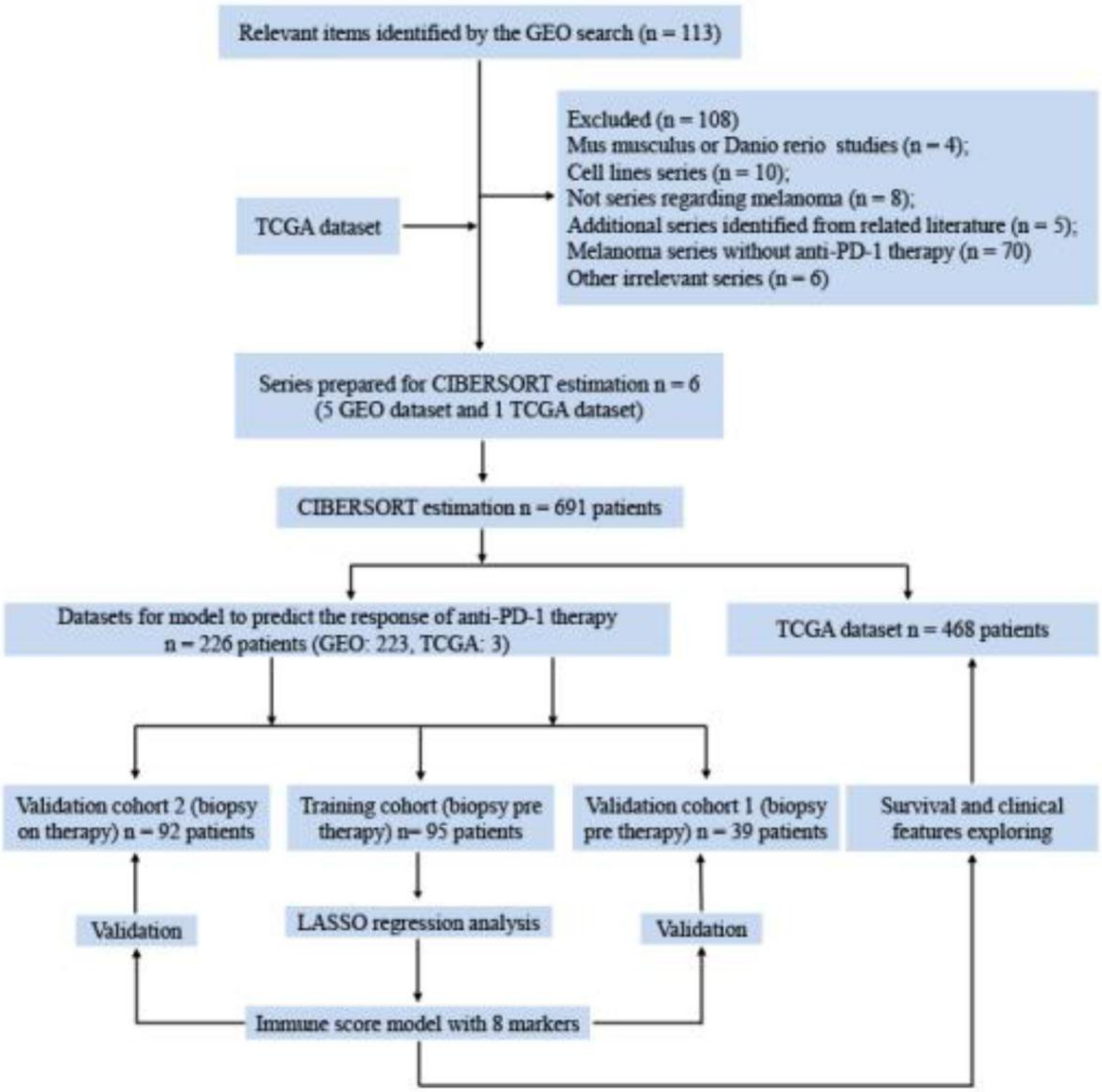

Supplementary Figure 1. CONSORT diagram. A total of 691 patients were enrolled in the analysis. GEO, Gene Expression Omnibus; TCGA, The Cancer Genome Atlas; CIBERSORT, Cell type Identification by Estimating Relative Subsets of RNA Transcripts; LASSO, least absolute shrinkage and selection operator; PD1, programmed death 1.

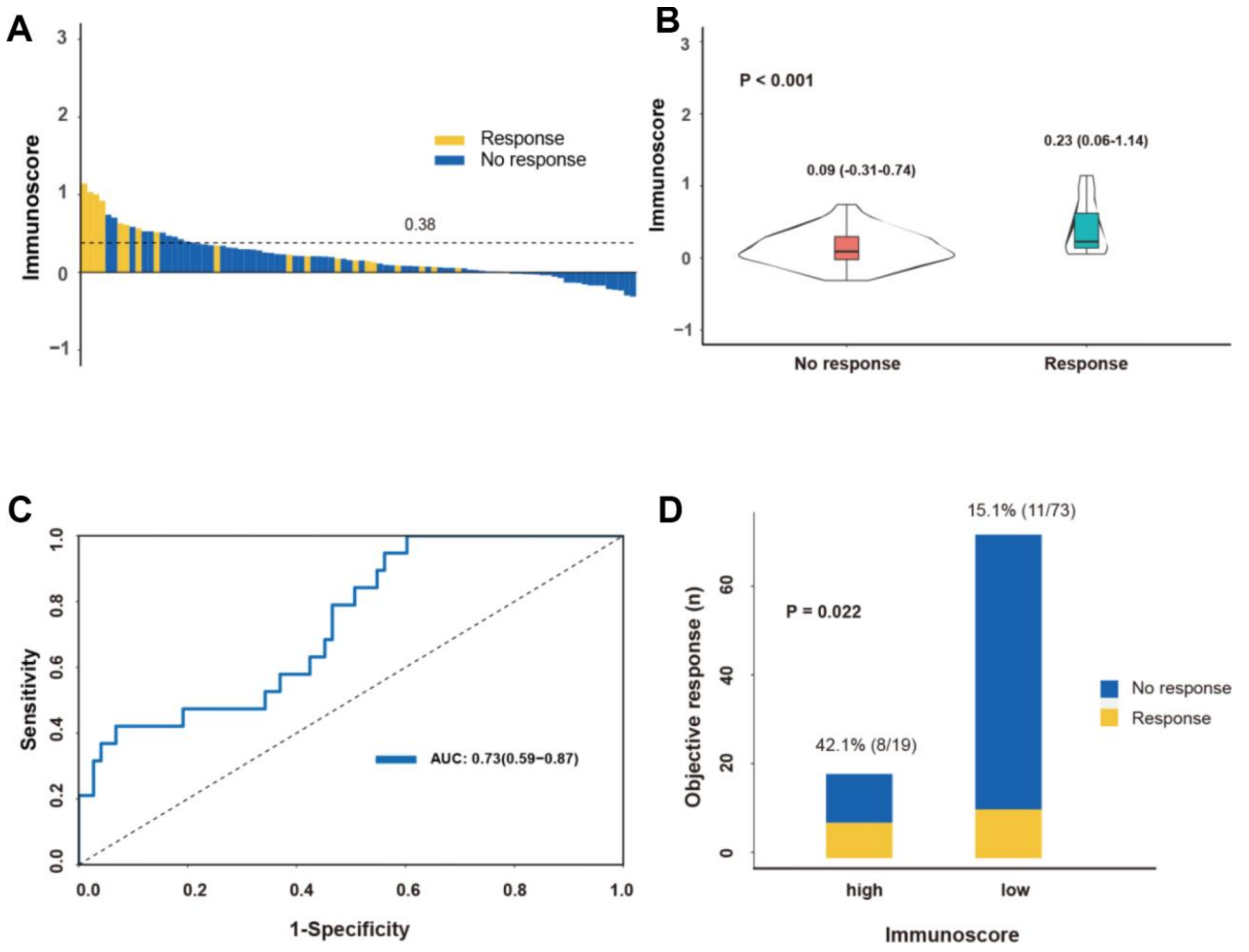

Supplementary Figure 2. Distribution of the immunoscore and response to anti-PD-1 therapy in the on-anti-PD-1 cohort. (**A**) Waterfall plots for distribution of the immunoscore and response status of individual patients. (**B**) Distribution of immunoscore in responders and nonresponders. The box plots inside the violin indicate the median value and interquartile range of immunoscore. We calculated the P-value with ANOVA test. (**C**) Receiver operating characteristic (ROC) curves of the immunoscore. The area under the ROC curve was 0·73. (**D**) Objective response rate between high- and low-immunoscore groups across the on-anti-PD-1 melanoma dataset. "On" indicates the biopsy during anti-PD-1 therapy. We calculated the P-value with the $\chi 2$ test.
