# Supplementary material for: Robust immunoscore model to predict the response to anti-PD1 therapy in melanoma
Source: Aging (Albany NY). 2019 Dec 3;11(23):11576–90. doi: 10.18632/aging.102556 (PMC6932919; doi:10.18632/aging.102556)
Supplement: Supplementary Table 1 [file aging-11-102556-s001..pdf]

Supplementary Table 1. Univariate and multivariate Cox regression analysis of immune score and clinical characteristics with overall survival in the TCGA cohort.

Variables	Univariate analysis		Multivariate analysis	
	HR (95% CI)	P-value	HR (95% CI)	P-value
Gender		0.301		
Female	1			
Male	1.16 (0.87-1.55)			
Age		< 0.001		0.001
< 60 year	1		1	
≥ 60 year	1.96 (1.43-2.68)		1.78 (1.27-2.50)	
TNM stage		0.001		0.003
I/II	1		1	
III/IV	1.63 (1.21-2.20)		1.58 (1.17-2.13)	
Immune score		0.016		0.002
Low	1		1	
High	0.72 (0.55-0.94)		0.63 (0.47-0.85)	

P-values were calculated with the two-sided log-rank test; TCGA, the Cancer Genome Atlas; HR, hazard ratio; CI, confidence interval.
